# One Dimensional Graphitic Carbon Nitrides as Effective Metal-Free Oxygen Reduction Catalysts

**DOI:** 10.1038/srep12389

**Published:** 2015-07-23

**Authors:** Muhammad Tahir, Nasir Mahmood, Jinghan Zhu, Asif Mahmood, Faheem K. Butt, Syed Rizwan, Imran Aslam, M. Tanveer, Faryal Idrees, Imran Shakir, Chuanbao Cao, Yanglong Hou

**Affiliations:** 1 Research Centre of Materials Science, Beijing Institute of Technology, Beijing 100081, China; 2 Department of Materials Science and Engineering, College of Engineering, Peking University, Beijing 100871 China; 3 Department of Electronics and Key Laboratory for the Physics and Chemistry of Nano devices, Peking University, Beijing 100871, China; 4 Sustainable Energy Technologies (SET) center building No 3, Room 1c23, College of Engineering, King Saud University, PO-BOX 800, Riyadh 11421, Kingdom of Saudi Arabia

## Abstract

To explore the effect of morphology on catalytic properties of graphitic carbon nitride (GCN), we have studied oxygen reduction reaction (ORR) performance of two different morphologies of GCN in alkaline media. Among both, tubular GCN react with dissolved oxygen in the ORR with an onset potential close to commercial Pt/C. Furthermore, the higher stability and excellent methanol tolerance of tubular GCN compared to Pt/C emphasizes its suitability for fuel cells.

A global energy crisis due to significant increase in industrial activities is a great challenge for future science. The depletion of fossil fuels is driving scientists to develop renewable and highly efficient methods for energy production and storage such as fuel cells, supercapacitors and lithium batteries which have proved to be favorable candidates in this aspect^1^,^2^,^3^,^4^,^5^,^6^,^7^. Among these, fuel cell got tremendous attentions of researchers due to its high efficiency and environment friendly nature. Fuel cells normally produce current by the electrochemical catalytic oxidation of a fuel, like ethanol, hydrogen or methanol at the cell cathode^4^,^5^,^6^,^7^,^8^,^9^. Oxygen reduction reaction (ORR) considered as a key factor in order to investigate the performance of fuel cell. Therefore, efficiency of ORR strongly depends on the catalyst used^8^,^9^,^10^. Nobel metals like Pt have been extensively studied as catalysts for ORR but high price, rare abundance, low stability, rapid degradation against carbon monoxide impurity, sluggish ORR kinetics needs high Pt loading to achieve acceptable power density and fuel crossover effect are the main hurdles in their practical applications^8^,^9^,^10^. Furthermore, Pt-based catalysts usually suffer from particle dissolution, surface oxide formation, and aggregation in alkaline electrolytes^9^,^10^. In contrast, non-precious transition metals, metal oxides, chalcogenides and their composites with carbon materials bring an advantageous improvement in the stability and crossover effect of fuels^11^,^12^,^13^,^14^. Despite wide abundance and good reaction kinetics, metal-based electrode materials still have several disadvantages, such as poor durability in strong acidic or basic environment, relatively high cost and detrimental environmental effects^11^,^12^,^13^,^14^,^15^. Thus, for the realization of fuel cells, it is necessary to develop stable metal-free ORR catalysts. Therefore, development of high performance, low cost and environment benign metal free catalysts are of great importance for fuel cells.

In this regard carbon based materials have been explored as active electrocatalysts. Introduction of heteroatoms such as nitrogen/sulphur/phosphorous in the carbon structure has also lead to significant improvements in electrocatalytic performances of carbonaceous materials such as carbon nanotubes (CNTs) and graphene^15^,^16^. Heteroatom-doping can enhance the electron-donor ability and improve their conductivity and catalytic properties by breaking the density of state (DOS) and electroneutrality of graphitic electronic cloud^16^,^17^,^18^. Among different heteroatom-doping, nitrogen-doping brings much improved results due to its higher electronegativity that cause net positive charge on the carbon atoms in its vicinity^19^,^20^. Thus, several nitrogen-doped carbon nanostructures such as graphene, CNTs and ordered mesoporous carbon have been studied for ORR that shows good catalytic activity^19^,^20^,^21^,^22^. However, a rational design of structure and morphology is required to improve the catalytic performance of these carbon-based materials. Thus, the introduction of graphitic carbon nitride (GCN) is a key solution to these issues^19^,^20^,^21^,^22^. The GCN has high nitrogen content, easily tuneable structure, cost effectiveness and environment friendly nature, which are potentially appropriate for ORR^23^,^24^,^25^,^26^. But the template based synthesis to develop its different morphology is disadvantageous as strong acids are needed to remove or etch the template^13^,^23^,^24^,^25^,^26^. Thus, template-free synthesis with unique one-dimensional (1D) nanostructures is highly required to improve the performance and its realization as cathode in fuel cells. The 1D nanostructures of GCN have large aspect ratio, high surface area, large number of active sites and short diffusion path for electrons and electrolyte/ions that are favourable for high performance as ORR catalyst in comparisons to previously reported bulk or powder carbon nitride (CN)^23^,^24^,^25^,^26^.

Herein, we present the template-free synthesis of 1D graphitic carbon nitride (GCN) in the form of nanofibers (GCNNF) and tubular (TGCN) as shown in Fig. 1. The products showed high surface area and high nitrogen contents that increase the wettability of the electrode and enhance the active sites which improve the overall performance, respectively. It was found that tubular structures exhibit higher ORR activity than nanofibers because of the availability of more active sites, higher surface area which increases electrolyte-electrode contact, highly accessible active sites and shorter diffusion path. Furthermore, GCNs followed the two and four electron pathway in the alkaline media with an onset potential close to that of commercial Pt/C. The stability of the GCNs is higher than that of Pt/C and exhibit excellent methanol tolerance. It is worth mentioning that morphology based studies of GCN will provide an understanding to manipulate the various materials to achieve maximum catalytic performance.

## Results

**Figure 1:**
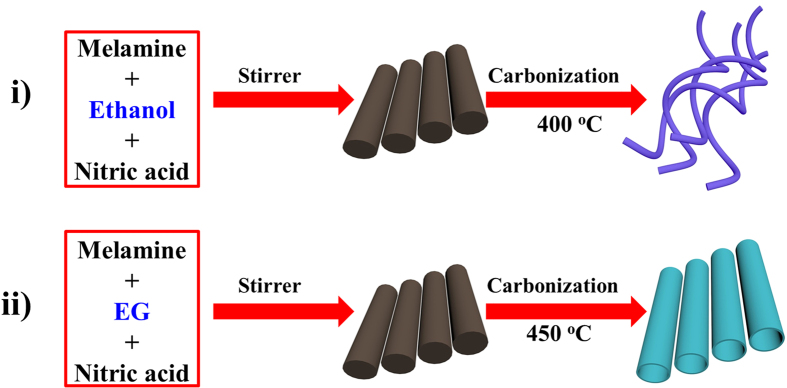
Schematic illustration of synthesis method for GCNNF and TGCN.

The structural analysis of GCNNF and TGCN were done using X-ray diffraction (XRD) spectroscopy as shown in Fig. 1a. XRD patterns of both samples clearly show the single well-defined broad peak at 27.3° corresponding to graphitic phase of carbon nitride and recognized as g-(002) with inter-planar distance of *d* = 0.326. These peaks are originated due to the long-range inter-planar stacking of aromatic systems^14^,^23^. The morphological aspects of TGCN and GCNNF were characterized using scanning electron microscope (SEM). Figure 1b and S1 are representing the tubular structure of TGCN with average length of ~15 μm and diameter of ~0.8 μm. Figure 1c and S2 clearly depicts the fiber like structure of the GCNNF which grew randomly with average diameter of ~150 nm and length ~20 μm. In order to further confirm the tubular structure of TGCN, transmission electron microscope (TEM) of the TGCN has been performed as shown in Fig. 1d. From the TEM image, it is obvious that TGCN were grown with the smooth surface and well-defined interior tubular structure. Furthermore, TEM studies show that as-synthesized TGCN has highly porous tube walls, which can accelerate the efficient transfer of mass in the electrode.

**Figure 2:**
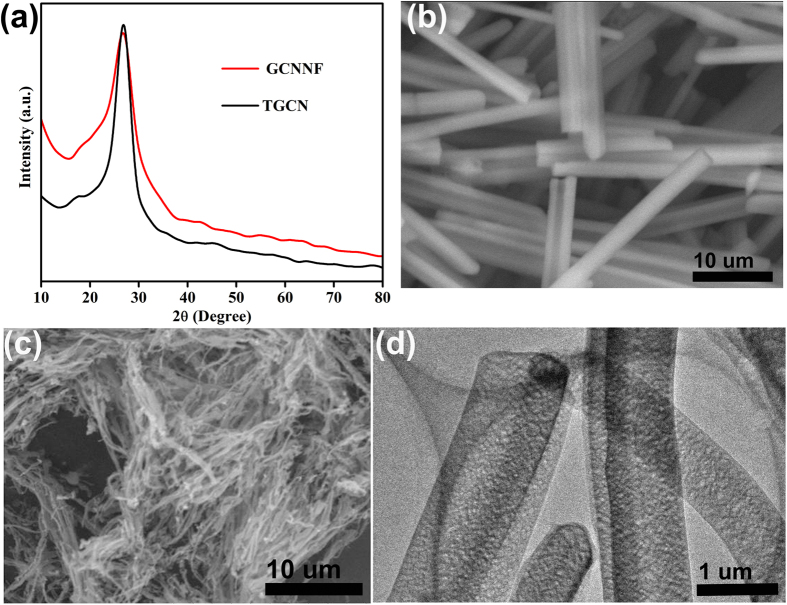
(**a**) XRD of TGCN and GCNNF, (**b**,**c**) SEM images of TGCN and GCNNF, respectively and (**d**) TEM image of TGCN.

Fourier transforms infrared spectroscopy (FTIR) was utilized to characterize the chemical bonds present in TGCN as shown in Figure S3. An absorption peak around 800 cm^−1^ is the strong indication of the heterocycles present in GCN, these peaks are due to the breathing mode of s-triazine and sp^2^ C = N^27^,^28^. The small peaks at 1060, 1260 and 1324 cm^−1^ shows the existence of single C–N bonds^28^. The peaks at 1367, 1382 and 1420 cm^−1^ are due to amorphous sp^3^ C–C bonds^27^,^28^. The peaks relevant to the stretching vibration modes of NH_2_ and NH groups are observed in the range of 3000–3500 cm^−1^ 27,28. Furthermore, to investigate the chemical compositions and the nature of chemical bonding of constituent elements in as-prepared GCN, X-ray photoelectron spectroscopy (XPS) was conducted as shown in Fig. 2a–d. The full scan spectrum of XPS analysis reveals the existence of core levels of C, O, and N in both GCN, which confirm the high purity of the as-prepared products (Figure S5 & S6). The de-convolution of C 1s peak (Fig. 2a,b) provides evidence for the presence of different chemical states of carbon in the as-synthesized products, the peak at 284.88 eV corresponds to *sp*^2^-hybridized carbon bonded to N atom in an aromatic ring, whereas second peak at 288.18 eV corresponds to C-N bonding in GCN. The nature of nitrogen species strongly effect the catalytic properties of carbon catalyst, thus de-convoluted N1s peaks of TGCN and GCNNF are presented in Fig. 2c,d, respectively. The peaks present at 400.8, 399.6, 398.6, 397.8 eV in TGCN and 400.6, 399.6, 398.8, 398 eV in GCNNF are corresponding to graphitic, pyrolic, amino and pyridinic nitrogen centers, respectively^2^,^29^. The percentage of each type of nitrogen is presented in Table S1, from where it is obvious that TGCN contains higher concentration of pyridinic nitrogen while GCCNF contains little higher concentration of graphitic nitrogen. The existence of large concentrations of pyridinic nitrogen and amino groups along with oxygen functionalities correspond to the better performance as cathodic ORR electrocatalysts. It is worth noting that the presence of graphitic and amino nitrogen determines the onset potential and electron transfer number in ORR process, while the enhanced oxygen reduction current density is ascribed to the total content of graphitic and pyridinic N.

**Figure 3:**
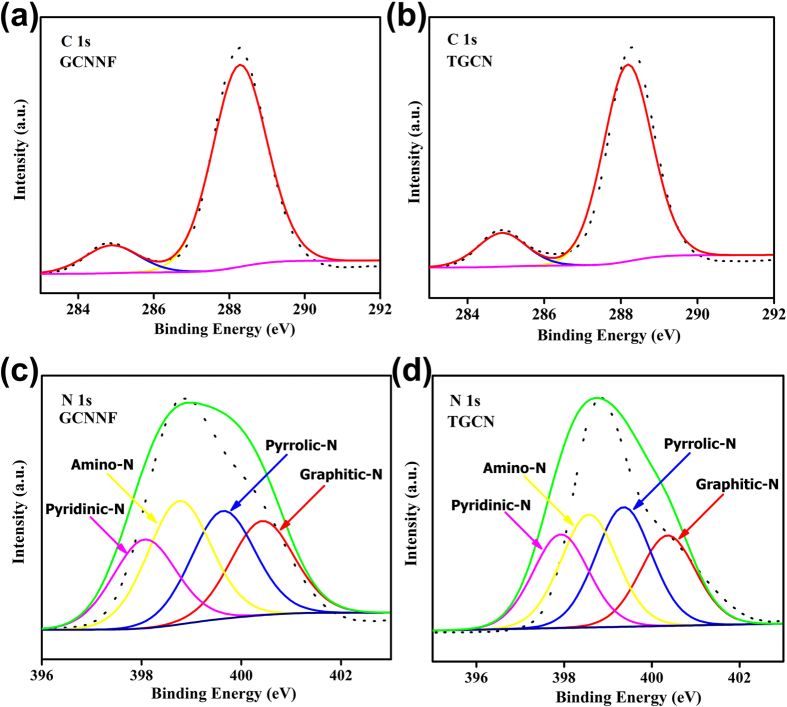
(**a,b**) High resolution C1s spectrums of GCNNF and TGCN, respectively, (**c,d**) High resolution N1s spectrums of GCNNF and TGCN, respectively.

The exposed surface of a material determines its ability to catalyse the reaction thus surface area measurements of TGCN and GCNNF were determined through Brunauer–Emmett–Teller (BET) method in the relative pressure range of 0–1. The measured surface area of TGCN is 32.27 m^2^/g that is higher than that of GCNNF (12.96 m^2^/g) as shown in Fig. 3a, The higher surface area of TGCN shows that it has large exposed surface with higher active sites to catalyse the oxygen in ORR process and provides large space to accommodate more charge storage sites. The elemental mapping analysis of TGCN was done using scanning transmission electron microscope (STEM) that confirms the spatial distribution of carbon, nitrogen, and small amount of oxygen which are consistent with XPS results that can be seen in Fig. 3b–e. Raman spectroscopy was utilized to further confirm the structure of carbon nitrides (TGCN and GCNNF) as shown in Figure S4. Where the broad peak appearing in the 1250–3000 cm^−1^ region are associated with the overlap of several of N-related peaks (N = C = N, 1400 cm^−1^; –CN, 1600 cm^−1^; atmospheric stretching vibration, 2300 cm^−1^)^30^.

**Figure 4:**
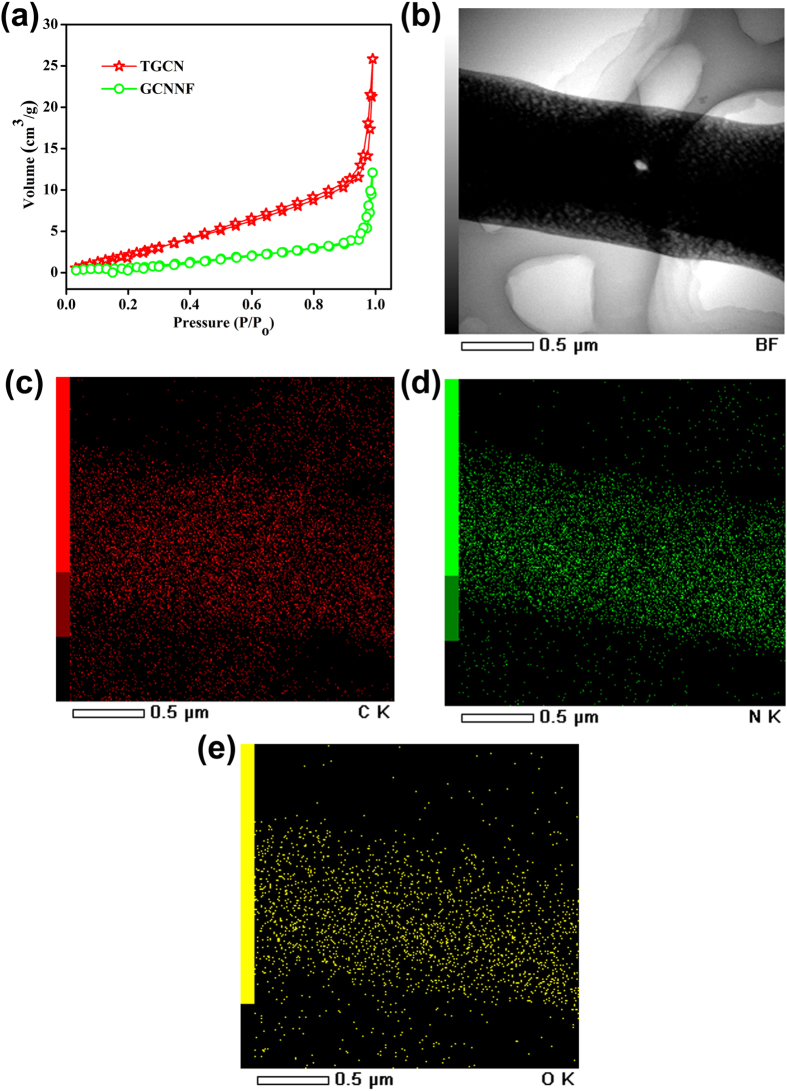
(**a**) N_2_ adsorption-desorption isothermal curves of TGCN and GCNNF, (**b**) STEM image of TGCN, (**c**) Carbon, (**d**) Nitrogen and (**e**) Oxygen distribution mapping of TGCN.

The ORR electrocatalytic activity of TGCN was investigated through loading the active materials on glassy carbon electrode for cyclic voltammetry (CV) in O_2_ saturated 0.1M KOH aqueous solution as shown in Fig. 4a. The CV of TGCN in O_2_ saturated solution clearly revealed its ORR catalytic activity with distinctive ORR onset potential around −0.15 V *vs.* Ag/AgCl and the peak current at the potential of −0.39 V *vs.* Ag/AgCl. The onset potential is calculated where 10% of the current value is achieved at the peak potential. Rotating disk electrode (RDE) measurements are further executed to scrutinize the ORR activity and kinetics of the TGCN and GCNNF 1 D nanostructures in O_2_ saturated 0.1 M KOH solution. Linear sweep voltammograms (LSV) for TGCN and GCNNF were recorded at 1600 rpm and compared with commercial Pt/C, as shown in Fig. 4b. The onset potential for TGCNF and CNNF were found to be ca. −0.15 and −0.14 V, respectively in comparison to commercial Pt/C (−0.02). The slightly better onset potential of GCNNF than TGCN is attributed to presence of higher concentration of graphitic and amino nitrogen but the higher current density of TGCN is due to higher concentration of pyridinic nitrogen (Table S1), large surface area and tubular morphology which provides large adsorption of oxygen and large active sites for the reduction of oxygen. Although, the current density is based on the concentration of oxygen and its reduction on the electrode-electrolyte interface but here when both samples (GCNNF and TGCN) were tested under same condition higher current density was found for TGCN because of the aforementioned reasons^29^. A better performance of both 1D nanostructures is based on the presence of higher content of nitrogen that disturbed the electronic structure of graphitic carbon and its DOS resulting in higher active sites with better performance. Furthermore, the interior tubular structure of TGCN shortened the diffusion path by providing dual pathway (from inside and outside of the tube) for diffusion and increased the electrolyte-electrode contact area. As a result, better catalytic activity with higher current densities was observed for TGCN. Furthermore, presence of oxygenated groups on the surface GCN nanostructures (as witnessed by XPS) further reduces the charge transfer resistance by providing more active sites on the surface of the electrode^5^.

In addition, LSVs were also recorded for TGCN and GCNNF from 400 to 2500 rpm to explore the exact kinetic parameters including electron transfer number (*n*) and kinetic current density (*J*_*K*_) using Koutecky–Levich (K-L) equations, shown in supporting information. It is worth noting that with increasing rotation speed the current density is increased for both 1D nanostructures due to availability of large diffused oxygen^4^. The calculated current densities at different potentials and rotation speeds are presented in the Fig. 4c,d for TGCN and GCNNF, respectively. From the current density values it is noted that the TGCN shows larger current densities as compared to GCNNF because of its tubular structure that shortened the diffusion pathways for dissolved oxygen and provides large active sites for its reduction. However, in case of GCNNF only surface reaction was observed that also limit the reaction transfer kinetics that was further supported by *n* calculations.

From the *n* calculation (according to K-L equation presented in supporting information) it is also concluded that GCNNF only possess two electron transfer process throughout all potential range. In contrast to GCNNF, TGCN shows two and four electron transfer processes per oxygen molecules (Figure S8 and S9). The higher n values and larger current density of TGCN with superior onset potential emphasize that tubular structure is more feasible for higher performance as electrode of ORR rather than fibrous one. Furthermore, an interesting phenomenon was observed that higher *n* value is found as potential moves towards negative^5^. Further, *n* calculation demonstrate that in case of GCNNF, formation of intermediate product (peroxide, H_2_O_2_) is found by the partial oxidation of oxygen as it shows transfer of two electron that is insufficient for complete reduction of oxygen instead of four electron that is the desired *n* value for better performance of fuel cells. But in case of TGCN ORR reaction proceeds through transfer of four electrons that proves inhibition of intermediate products and carried out the complete reduction of oxygen to water.

**Figure 5:**
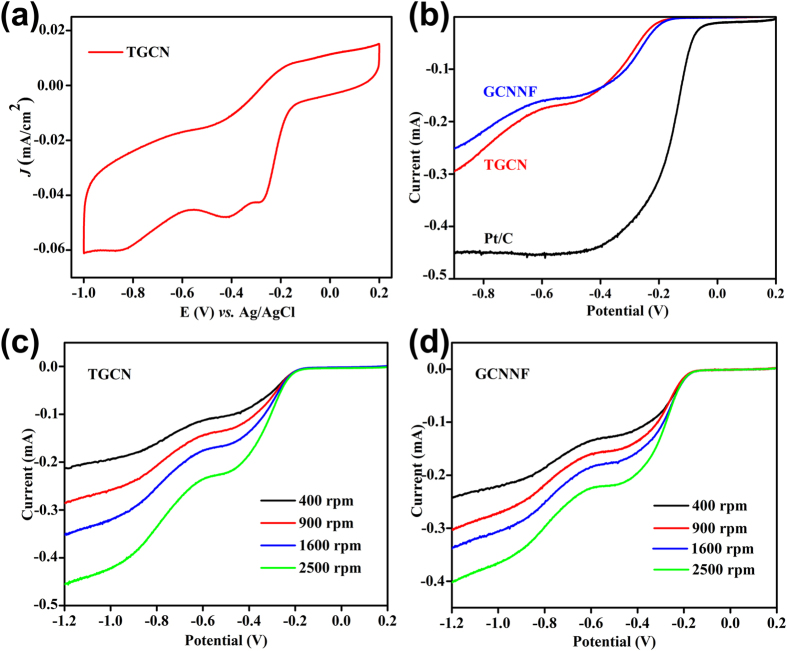
(**a**) CV of TGCN in 0.1 M KOH solution at scanning rate of 100 mV/s (**b**) LSV curves of TGCN, GCNNF and Pt/C electrodes in an O_2_ saturated 0.1 M KOH solution at a scanning rate of 10 mV/s and a rotation speed of 1600 rpm (**c,d**) RDE curves of TGCN and GCNNF electrode in an O_2_ saturated 0.1 M KOH solution with different rotation speeds at a scanning rate of 10 mV/s respectively.

**Figure 6:**
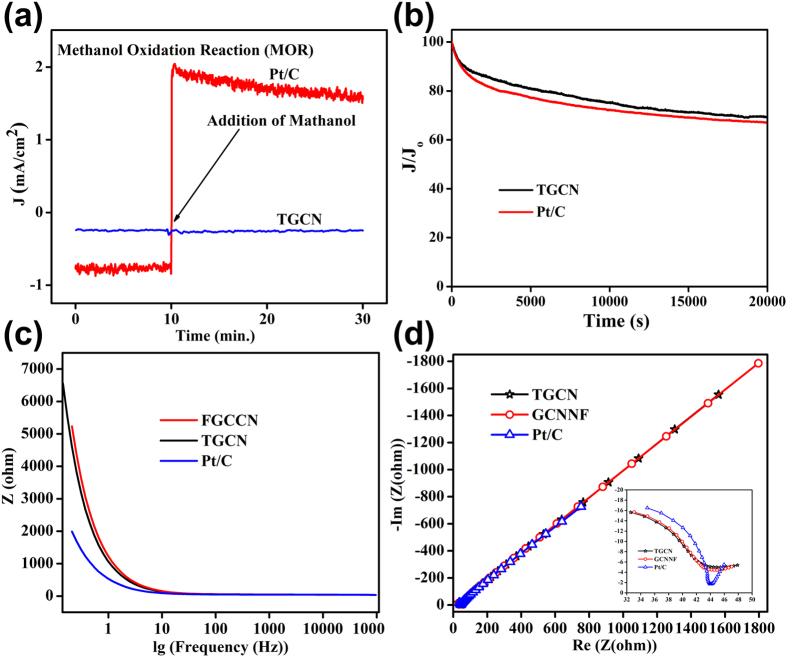
(**a**) Methanol crossover effect test of PG and Pt/C upon addition of 3 M methanol after about 10 min in an O_2_ saturated 0.1 M KOH solution at −0.25 V. (**b**) Current-time response of TGCN and Pt/C electrodes at −0.5 V in an O_2_-saturated 0.1 M KOH at a rotation speed of 1600 rpm. Bode (**c**) and Nyquist (inset is the enlarge spectrum in lower frequency) (**d**) spectra of TFCN, FGCCN and Pt/C under a sine wave of 5.0 mV amplitude in the frequency range of 100 kHz to 10 mHz.

Moreover, for the sake of comparison the TGCN 1D nanostructure and commercial Pt/C catalysts are further tested for methanol crossover *via* chrono-amperometric responses at the potential of −0.25 V in O_2_ saturated 0.1 M KOH electrolyte (Fig. 5a). Because one of the major hurdles in long cyclic performance of commercial Pt/C as cathodic ORR catalysts is that its performance is drastically affected with the addition of methanol. It is observed that the current decayed in similar fashion for the TGCN 1D nanostructure and commercial Pt/C catalysts before methanol addition. However, after the addition of 3 M methanol a drastic change in current decay is observed for commercial Pt/C catalyst shows a rapid drop in performance and initiation of methanol oxidation reaction (MOR), while that of the TGCN 1D nanostructure shows stable amperometric response even after addition of 2 wt% methanol. This indicates that the TGCN structure owns excellent methanol tolerance, which strongly emphasizes its use for practical applications. Furthermore, the durability of the TGCN 1D nanostructure was examined in 0.1M KOH for 20,000 s and it is found that the TGCN 1D nanostructure is more stable in basic medium than the commercial Pt/C (Fig. 5b & S10). The higher values of relative current for the TGCN 1D nanostructure than Pt/C after 20,000 s confirms its superior stability than Pt/C. Such high durability of the TGCN 1D nanostructure is due to its unique tubular structure that provides higher contact area between electrode and electrolyte and larger active sites for ORR. Therefore, the TGCN 1D structure synthesized in present work is a promising electrocatalyst for ORR in fuel cells. Although the onset potential of TGCN 1D nanostructure is slightly lower than Pt/C but its high stability and very good methanol tolerance emphasize its dominance over Pt/C and prove it to be a better contestant in the ORR. Furthermore, to investigate the reason for better performance of TGCN than GCNNF, also over all better stability with improved current density, electrochemical impedance spectroscopy (EIS) was carried out. The Bode (Fig. 6c) and Nyquist (Fig. 6d) results confirm that TGCN exhibits much better overall conductivity than that of GCNNF; therefore, the enhanced ORR performance both in current density and electron transfer number is attributed to its lower resistance to electron and faster mass transport. However, the enlarge Nyquist spectrum (inset of Fig. 6d) shows that Pt/C bears lower charge transfer resistance (*R*_*c*t_) than both of the GCN structures but the more straight inclined line in the higher frequency region proved that GCN offers much lower resistance (Warburg impedance (W)) for mass transport and higher wettability of the electrode.

## Discussion

The growth mechanism of GCN is described in Fig. 1, from where it is obvious that polyaddition and polycondensation reactions together in a continuous process built the sheets of GCN that can provides the different shapes depending on the reaction medium and conditions. In chemistry point of view initially activation of melamine to heptazine or cyamelurine was done by eliminating ammonia through nitric acid treatment. The condensation reaction among heptazine or cyamelurine unites built a polymeric network that results in GCN at higher synthesis temperature. Although the melamine goes to sublimation at higher temperature but here strong covalent bonding among these reacting sub unites made the melamine stable at higher temperature and results in the formation of GCN. After activation of melamine by nitric acid results in heptazine, which goes to polymerization process and form the melon and melon sheets. The melon sheets results in the fibrous morphology if the reaction medium is polar (ethanol), thus introduction of ethanol in the reaction system results in GCNNF structure in the present study. The strong hydrogen bonding or Van der Waals forces are strongly held these sheets to construct the various morphologies of GCN. Furthermore, by changing the reaction solvent with ethylene glycol (EG) monomers instead of ethanol can construct the tubular structure of TGCN simply introducing extra carbon during polymerization process. Probably, when nitric acid breaks the electroneutrality of aromatic rings of melamine as mentioned above, the EG polymerize on the defects created by nitric acid to construct a tubular structure. The carbonization of melamine (after activation by nitric acid) by EG modifies the reaction products to build the tubular structure and stabilize the resulted tubular morphology of TGCN. Further EG also maintains the carbon contents in GCN to alleviate the phase of g-C_3_N_4_ and continuous feeding of carbon by EG during annealing results in highly stable tubular morphology of TGCN. In addition, the growth mechanism of the tubular structure is mainly based on controlled synthesis temperatures, disturbance of the electroneutrality that offers the large number of anchoring sites to EG which tends to force the melamine to build a unique tubular morphology. In fact, the movement of carbonated species from inside to outward during annealing process creates tubular structure. Usually, GCN based materials display symmetry similar to scaffolding tri-s-triazine hexagonal structure and inter grown domains exhibit graphite-like stacking in GCN. Furthermore, there are several reports that discussed about the active sites location and their catalytic mechanisms in nitrogen-doped carbon based materials but are still inconclusive might be partially due to unavailability of appropriate characterization method. However, it is well-known fact that heteroatom-doping such as nitrogen creates net positive charge by disturbing the DOS because of its higher electronegativity. Thus, these partially positive carbon atoms are the active sites in nitrogen-doped carbon materials where the reduction of oxygen occurs and can be determined by observing the adsorbed reaction products like hydroxyl groups after the catalysis process. It is believed that the pyridinic nitrogen especially located at the edges plays a critical role through larger influence on the electronic structure of the carbon atoms in its vicinity. In short, similar to nitrogen-doped CNTs/graphene, here improved catalytic activity is based on the simple electron accepting capability of nitrogen atoms from the adjacent carbon atoms which creates a net positive charge on the GCN structure that effectively attract electrons from the anode side. Further nitrogen-doping induces over all charge delocalization in GCN structure which effectively changes the chemisorption mode of O_2_ from end-on adsorption to a side-on adsorption, efficiently weakens the O–O bond and assists the ORR process. However, the microstructure or location of doped nitrogen atoms implies strong effect on the catalytic performance of nitrogen-doped carbon based materials such GCN, thus by developing different morphologies change location or vicinity of the nitrogen atoms *e.g.* twisted tubular structure of TGCN brings more nitrogen atoms at edge sides thus enhance the overall performance of TGCN than GCNNF.

In summary, we have successfully fabricated different 1D nanostructures of GCN (tubular and nanofibers) catalysts with excellent performance as electrode of ORR for fuel cells. Among these TGCN catalyst shows prominent ORR catalytic activity that is slightly lower than commercial Pt/C regarding reaction current density and onset potential. TGCN also showed high fuel crossover resistance and much better stability in comparison to the commercial Pt/C in alkaline medium. The extraordinary performance of TGCN corresponds to its unique structure and presence of higher nitrogen contents that provides larger surface area for oxygen diffusion and active sites for its reduction, respectively. Furthermore, these 1D nanostructures were simply synthesized using inexpensive melamine as a precursor through a facile template free method which gives a great promise for large scale production without further treatment to remove the template *via* strong acids. The morphology of the GCN can easily be tailored by making a slight change in the mentioned procedure that emphasizes the generalization of the devised synthesis method. All the aforementioned features make proposed 1D nanostructures of GCN promising and potential substitutes instead of expensive noble metal catalysts at cathodic ORR in the next generation methanol fuel cells.

## Methods

### Fabrication of TGCN and GCNNF

TGCN and GCNNF were synthesized according to our previously established methodologies. Typically TGCN was prepared by dissolving 1 g of melamine in 30 mL of ethylene glycol to make a saturated solution. Then 60 ml of 0.1M HNO_3_ was added and stirred for 10 min. Final product was washed with ethanol and dried at 60 °C for 12 h. At last, white color powder was obtained which was annealed at 450 °C for 2 h at heating rate of 10 ^°^C/min.

Similarly for GCNNF, 1 g of melamine was dissolved in 20 mL of ethanol and addition of, 60 mL of 0.2 M HNO_3_ was done with 10 min stirring. The obtained product was washed with ethanol and dried at 60 °C for 12 h. Afterward the final white color powder was annealed at 400 ^°^C for 2 h in furnace in alumina tube at heating rate of 10 °C/min.

### Characterization

The structure of the as-synthesized products was characterized by x-ray diffraction (XRD Philips X’Pert Pro MPD) with standard Cu-Kα radiation source, λ = 0.15418 nm in the 2*θ* range of 10–80°. Fourier transforms infrared (FTIR) spectroscopy by using Nicolet Avatar-370. The morphological characterization of the product was carried out using FEI Tecnai T20 transmission electron microscopy (TEM) and scanning electron microscopy (SEM, Hitachi S-4800). X-ray photoelectron spectroscopy (XPS) was carried out using a PHI Quantera II (ULVAC-PHI, Japan) XPS system with monochromatic Al-Kα excitation under a vacuum better than 10^−7 ^Pa. Scanning transmission electron microscopy (STEM) analysis was done using JEM-2100F. Surface area was determined using Beishide Instrument-ST, 3H-2000PS2 through Brunauer–Emmett–Teller (BET) equation in the relative pressure range of 0–1.

### Fabrication of electrode for ORR

Rotating-disk electrode (RDE) measurements were carried out by using a CHI 760C electrochemical workstation with a three-electrode system. Working electrode consisted of glassy-carbon (GC) (diameter 5 mm), a Pt foil was used as counter electrode and an Ag/AgCl with saturated KCl solution as reference electrode. Electrode was prepared by making the suspension of active materials with carbon (in ratio of 4:1) in ethanol (1 mg mL^−1^) under sonication. After sonication 10 μL of this solution was incorporated on the GC RDE using a micro-syringe, followed by dropping 5 μL of Nafion solution in ethanol (0.1 wt %) as a binder for the better adhesion of catalyst to electrode. Electrolyte is consisted of O.1M KOH aqueous solution.

### Calculation for Electron Transfer number

As the number of electron transfer (*n*) per oxygen molecules is very important to determine the efficiency of catalyst for ORR electrode. Thus, in order to calculate *n* per oxygen molecule for oxygen reduction at electrode we used the K-L equation given below.


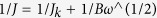


Here J is current density; *J*_*k*_ is kinetic limiting current densities and *ω* is electrode rotation rate. This equation was used to measure the *B* from the slope of K-L plots as given below:


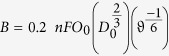


Here *n* is the number of electrons transferred per O_2_, F is the Faraday constant (F = 96485 Cmol^−1^), C_0_ is bulk concentration of O_2_, D_0_ is diffusion coefficient of O_2_ in 0.1 M KOH, and υ is a kinetic viscosity. The constant 0.2 was necessary when the rotation rate is expressed in rpm. By using the values C_0_ = 1.2 × 10^−6^ mol cm^−3^, D_0_ = 1.9 × 10^−5^ cm^2^ s^−1^, and υ = 0.01 cm^2^ s^−1^, *n* was calculated.

## Additional Information

**How to cite this article**: Tahir, M. *et al.* One Dimensional Graphitic Carbon Nitrides as Effective Metal-Free Oxygen Reduction Catalysts. *Sci. Rep.* **5**, 12389; doi: 10.1038/srep12389 (2015).

## Supplementary Material

Supplementary Information
