# Supplementary material for: One Dimensional Graphitic Carbon Nitrides as Effective Metal-Free Oxygen Reduction Catalysts
Source: Sci Rep. 2015 Jul 23;5:12389. doi: 10.1038/srep12389 (PMC4511877; doi:10.1038/srep12389)
Supplement: Supplementary Information [file srep12389-s1.docx]

Supporting Information

One Dimensional Graphitic Carbon Nitrides as Effective Metal-Free Oxygen Reduction Catalysts

*Muhammad Tahir^a,‡^, Nasir Mahmood^b,‡^, JinghanZhu^b^, Asif Mahmood^b^,Faheem K. Butt^a^,Syed Rizwan^c^, Imran Aslam^a^, M. Tanveer^a^, Faryal Idrees^a^, Imran Shakir^d^, ChuanbaoCao^a,*^,Yanglong Hou^b,*^*

[*,^a^]Prof. C. Cao, M. Tahir, F.K. Butt, I. Aslam, M. Tanveer, F. Idrees

Research Centre of Materials Science, Beijing Institute of Technology,

Beijing 100081, China

E-mail: [cbcao@bit.edu.cn](mailto:cbcao@bit.edu.cn)

[*,^b^]Prof. Y. Hou, N. Mahmood, J. Zhu, A. Mahmood

Department of Materials Science and Engineering, College of Engineering,

Peking University, Beijing 100871 China
E-mail: [hou@pku.edu.cn](mailto:hou@pku.edu.cn)

[^c^]Prof. S. Rizwan

Department of Electronics and Key Laboratory for the Physics and Chemistry of Nanodevices,

Peking University, Beijing 100871, China

[^d^] I. Shakir

Sustainable Energy Technologies (SET) center building No 3, Room 1c23, College of Engineering, King Saud University, PO-BOX 800, Riyadh 11421, Kingdom of Saudi Arabia

[^‡^]These authors contributed equally





**Figure S1**.SEM image of TGCN.


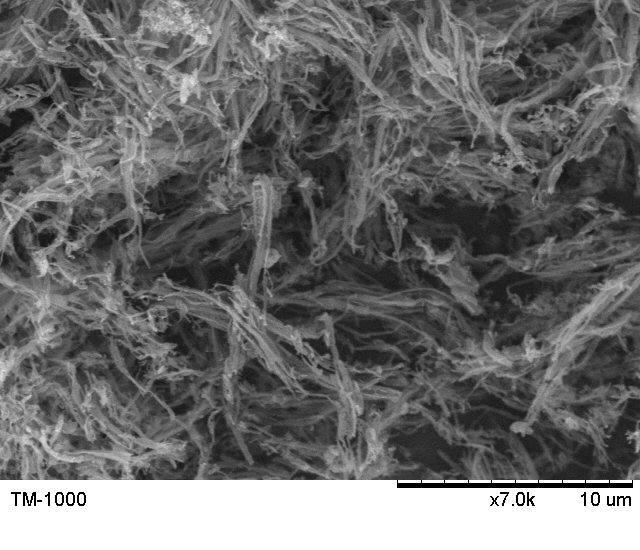


**Figure S2**.SEM image of GCNNF.


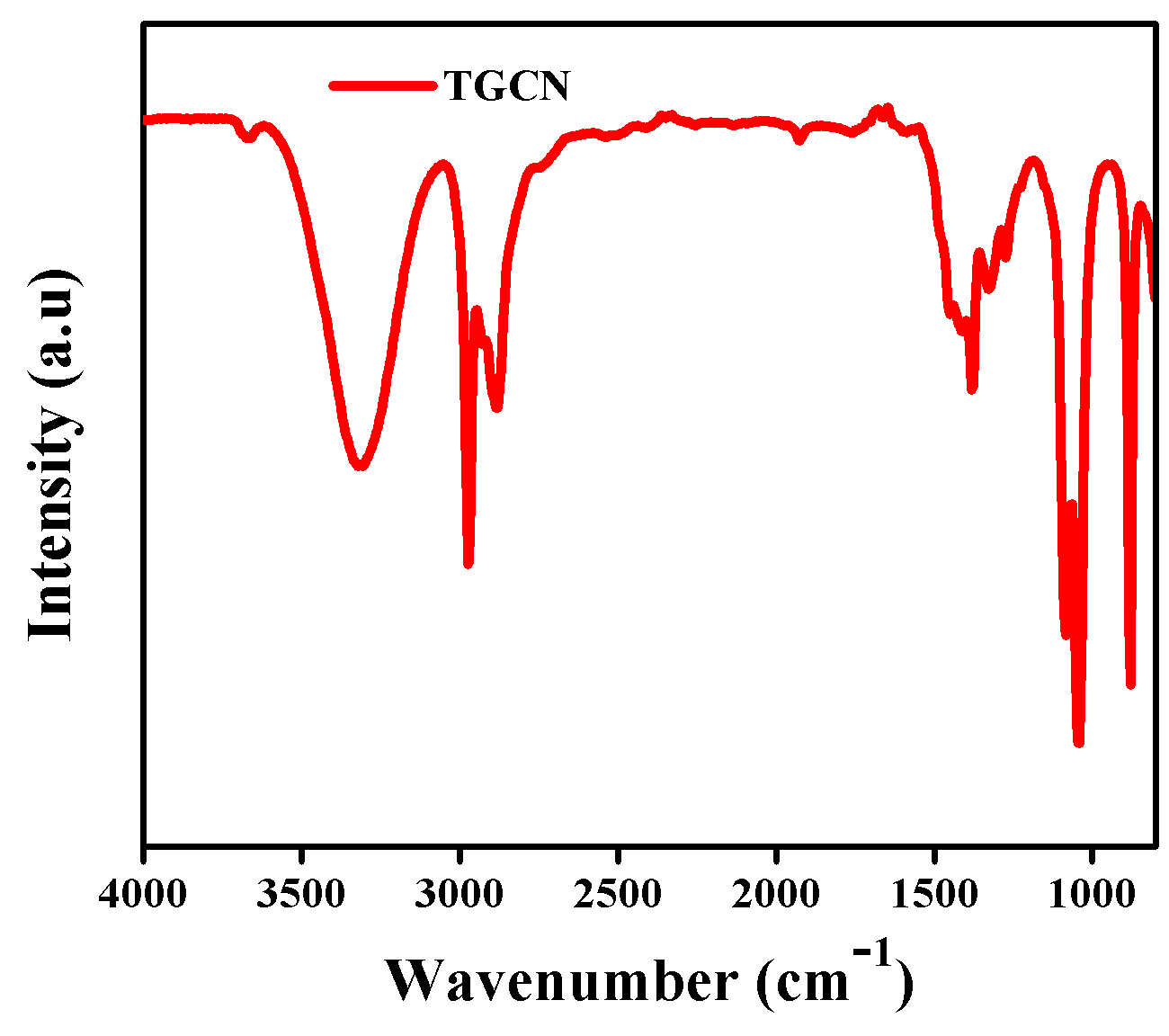


**Figure S3**. FTIR spectrumof TGCN.


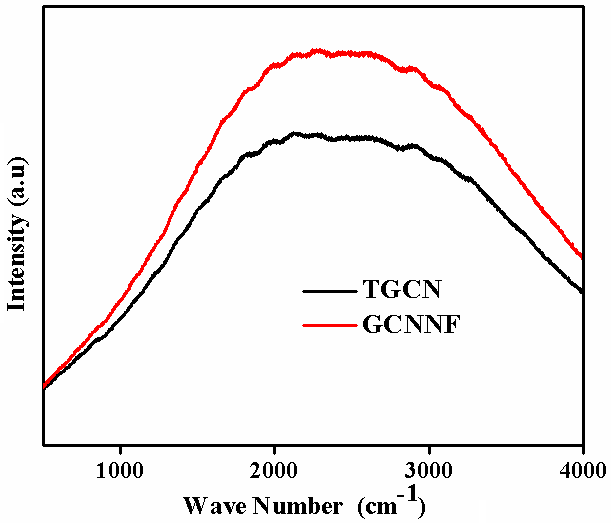


**Figure S4**.RAMAN spectrum of TGCN and GCNNF.


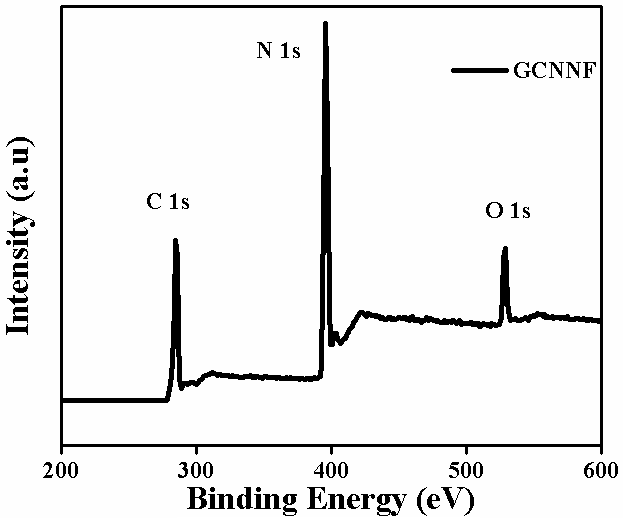


**Figure S5**.XPS spectra of GCNNF.


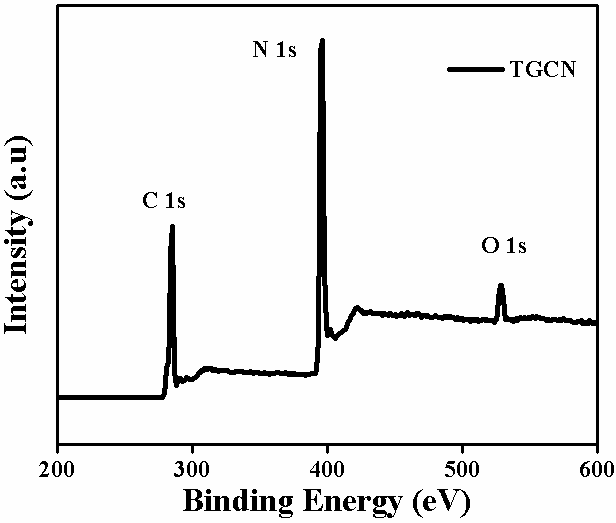


**Figure S6.**XPS spectra of TGCN.


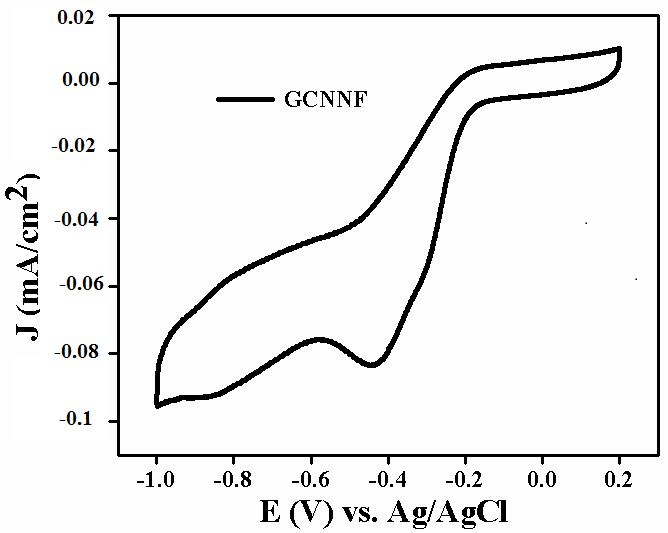


**Figure S7.** CV of GCNNF in0.1 M KOH solution at scanning rate of 100 mV/s.

**
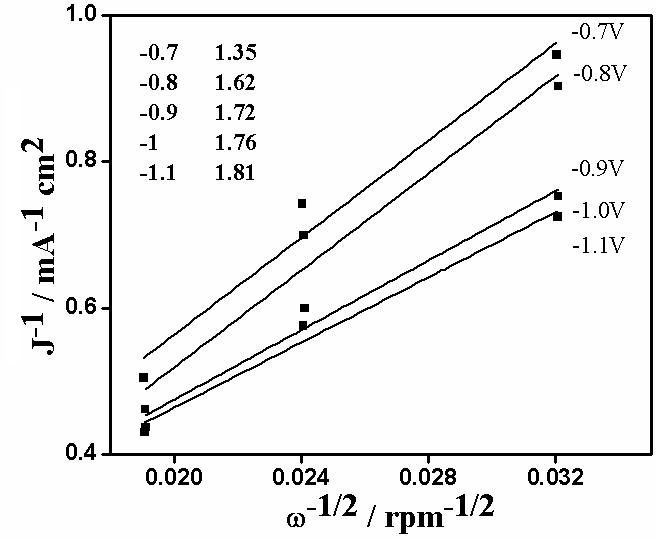
**

**Figure S8**.K–L plots of *J^-1^* versusω^-1/2^at different electrode potentials derived from RDE measurements for TGCN.

**
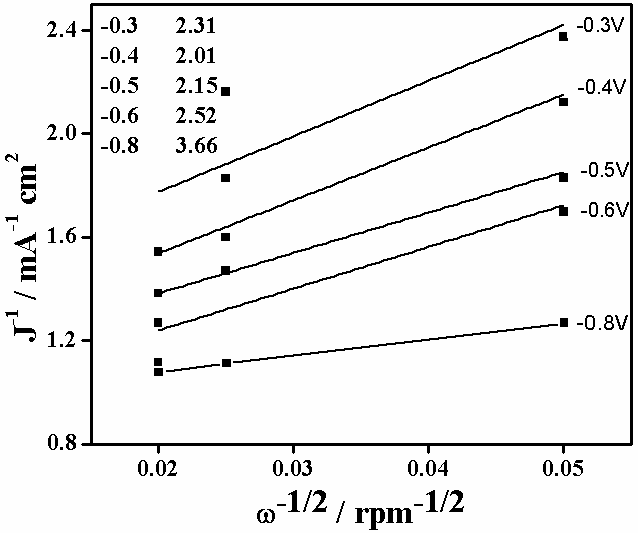
**

**Figure S9**. K–L plots of *J^-1^* versusω^-1/2^at different electrode potentials derived from RDE measurements for GCNNF.

**

**

**Figure S10.** Current-time response of TGCN electrode at -0.28 V in an O_2_-saturated 0.1 M KOH at a rotation speed of 1600 rpm

**Table S1:** The percentage of different type of nitrogen in GCCNFand TGCN calculated from XPS analysis.

| Sample  Name | Pyridinic  N% | Amino  N% | Pyrrolic  N% | Graphitic  N% |
| --- | --- | --- | --- | --- |
| GCNNF | 21.44 | 29.8 | 26.02 | 22.74 |
| TGCN | 22.47 | 27.1 | 28.71 | 21.71 |
